# A Comprehensive Review of Types, Properties, Treatment Methods and Application of Plant Fibers in Construction and Building Materials

**DOI:** 10.3390/ma15124362

**Published:** 2022-06-20

**Authors:** Muhammad Nasir Amin, Waqas Ahmad, Kaffayatullah Khan, Ayaz Ahmad

**Affiliations:** 1 Department of Civil and Environmental Engineering, College of Engineering, King Faisal University, Al-Ahsa 31982, Saudi Arabia; kkhan@kfu.edu.sa; 2 Department of Civil Engineering, COMSATS University Islamabad, Abbottabad 22060, Pakistan; waqasahmad@cuiatd.edu.pk; 3 MaREI Centre, Ryan Institute and School of Engineering, College of Science and Engineering, National University of Ireland Galway, H91 HX31 Galway, Ireland; a.ahmad8@nuigalway.ie

**Keywords:** sustainable materials, plant fibers, green materials, scientometric analysis, construction materials’ applications

## Abstract

Sustainable development involves the usage of alternative sustainable materials in order to sustain the excessive depletion of natural resources. Plant fibers, as a “green” material, are progressively gaining the attention of various researchers in the field of construction for their potential use in composites for stepping towards sustainable development. This study aims to provide a scientometric review of the summarized background of plant fibers and their applications as construction and building materials. Studies from the past two decades are summarized. Quantitative assessment of research progress is made by using connections and maps between bibliometric data that are compiled for the analysis of plant fibers using Scopus. Data refinement techniques are also used. Plant fibers are potentially used to enhance the mechanical properties of a composite. It is revealed from the literature that plant-fiber-reinforced composites have comparable properties in comparison to composites reinforced with artificial/steel fibers for civil engineering applications, such as construction materials, bridge piers, canal linings, soil reinforcement, pavements, acoustic treatment, insulation materials, etc. However, the biodegradable nature of plant fibers is still a hindrance to their application as a structural material. For this purpose, different surface and chemical treatment methods have been proposed in past studies to improve their durability. It can be surmised from the gathered data that the compressive and flexural strengths of plant-fiber-reinforced cementitious composites are increased by up to 43% and 67%, respectively, with respect to a reference composite. In the literature, alkaline treatment has been reported as an effective and economical method for treating plant fibers. Environmental degradation due to excessive consumption of natural resources and fossil fuels for the construction industry, along with the burning of waste plant fibers, can be reduced by incorporating said fibers in cementitious composites to reduce landfill pollution and, ultimately, achieve sustainable development.

## 1. Introduction

**Figure 1:**
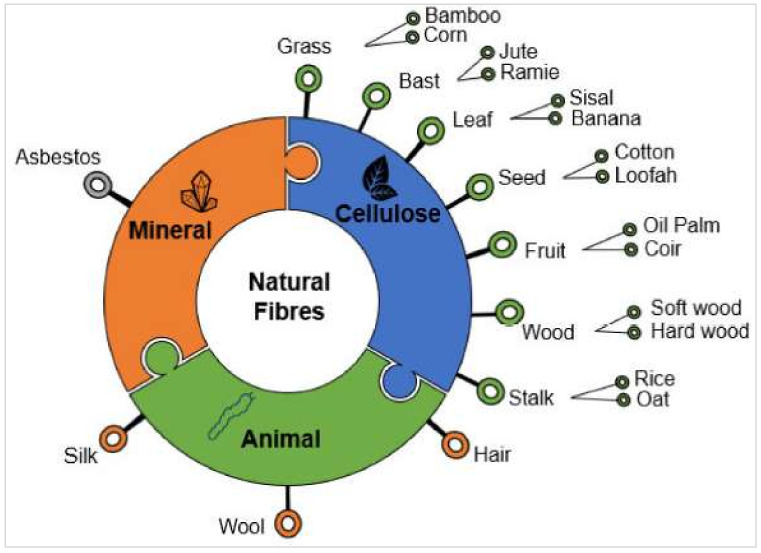
Schematic view of natural fibers’ classification [5].

The methodology of reduction in the environmental impact of any product is essentially the reconsideration of said impact throughout the whole life cycle, considering factors [1] such as the (1) production method, (2) development method, (3) packaging, (4) preservation, (5) usage, and (6) disposal and/or recycling. Potential retaliation from the customers may be faced by the designers in the event that they ignore environmental aspects during the design process. Keeping in mind the enhanced competition in the product market, the need to incorporate environmentally friendly materials is becoming the main basis of design today. An elongated piece of thread/hair-like continuous filament is called a fiber, whereas a fiber group that is twisted in the form of filament, thread, or rope is termed “fibers” [2,3,4]. These are highly beneficial as an element of composite materials. The sources of natural fibers are minerals, plants, and animals. Neto et al. [5] presented the broad classification of natural fibers into three main groups: plant (e.g., cellulose/lignocellulose), mineral, and animal fibers (Figure 1).

**Figure 2:**
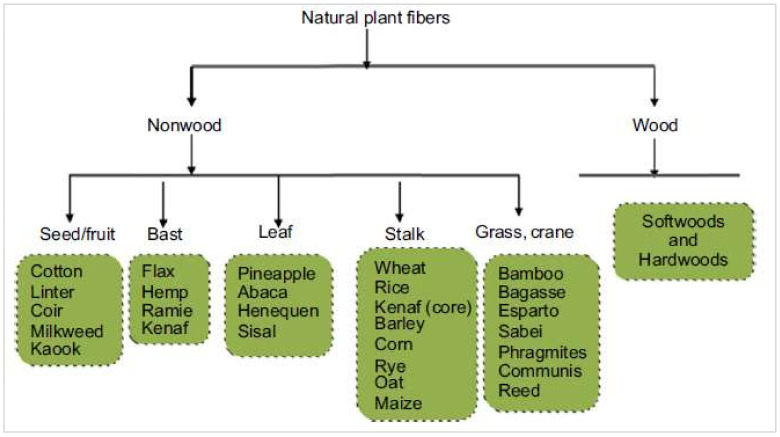
Natural fibers’ classification [14].

**Figure 3:**
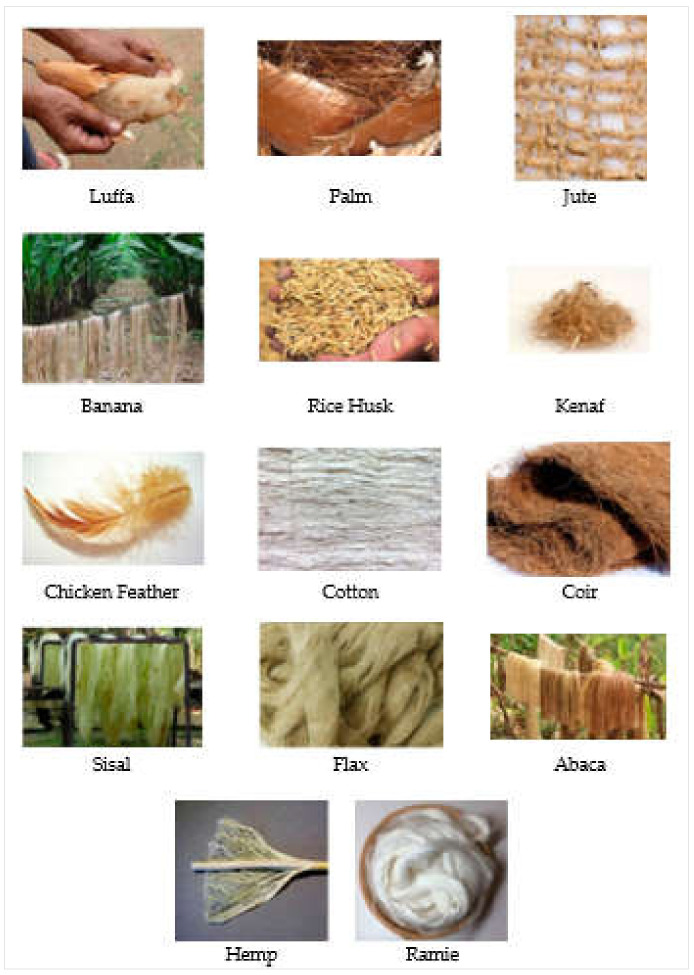
Natural fibers [52].

Accordingly, fibers are broadly classified into three categories: (1) natural plant fibers, (2) manmade fibers, and (3) synthetic fibers. The manmade and synthetic fibers have been studied by various researchers for their possible applications as construction and building materials [6,7,8,9,10]. Alyousef et al. [11] investigated waste-polypropylene-fiber-reinforced concrete for its possible application as an insulation material. However, natural fibers have some unique advantages as compared to manmade and synthetic fibers, such as low cost, environmental friendliness, and abundant resources [12,13]. Figure 2 depicts the natural plant fibers’ broader classification, as presented in [14]. Almost 2000 types of plant fibers are available globally, e.g., wheat straw, coir, palm, kenaf, sugarcane bagasse, cotton, bamboo, basalt/wool, flax, corn, hemp, hay, jute, henequen, ramie, sisal, banana, and pineapple leaf [15,16,17,18,19,20,21,22,23,24,25]. Some plant fibers are shown in Figure 3. Alyousef et al. [26] studied sheep-wool-fiber-reinforced composites under impact loading, and reported satisfactory outcomes. Plant fibers are gaining the attention of researchers in the construction material sciences field for the exploration of their potential to be used in composites, with the aim of sustainable development. In the past decade, the popularity of eco-friendly plant fibers has been considerably enhanced because of their favorable properties, including cheap and abundant availability, flexibility in handling and usage, low density, comparable mechanical characteristics, high strength-to-weight ratios, etc. [27,28,29,30,31,32,33,34]. However, properties such as low durability, poor bonding, increased water absorption, and comparatively worse thermal and mechanical properties with respect to artificial fibers, still provide much hindrance in practical applications of plant fibers [35,36,37,38,39]. Various efforts in terms of hybridization, incorporation of nanofillers, and treatment of fibers have been made to overcome said deficiencies of plant fibers. Alyousef et al. [40] applied the fiber hybridization technique for enhancing the durability of plant-fiber-reinforced concrete. Accordingly, in recent years, processed plant-fiber-reinforced composites have been considered for construction and building material applications [28,39,41,42,43,44,45,46,47,48,49,50,51].

Today, the key concern of modern development is sustainability. In pursuit of this, global warming due to swift ozone layer depletion because of environmental degradation is also a pressing issue. Environmental degradation is mainly caused because of extreme consumption of natural resources and fossil fuels. Hence, in pursuit of sustainable development, as per the sustainability goals defined by United Nations Development Programme (UNDP), the growing environmental pollution (the cause of ozone layer depletion) needs to be reduced. The burning of agricultural/plant wastes in subtropical and tropical areas is the main contributor to air/environmental pollution. Furthermore, the progressing consumption of natural resources and fossil fuels to cater for the needs of the construction industry also contributes towards environmental degradation. Therefore, the incorporation of different agricultural waste/plant fibers in various composites can play a significant role in the achievement of the UNDP sustainability goals. This incorporation would not only contribute towards a reduction in the overall costs of composites, it would also decrease the consumption of conventional materials, ultimately leading towards a reduction in the consumption of natural resources.

In pursuit of sustainable development, as per the UNDP sustainability goals, the environmental pollution that is depleting the ozone layer needs to be addressed. The selection of materials for manufacturing and associated design of sustainable materials plays a significant role in the construction industry. The composites are tailor-made materials that exhibit variable properties dependent on the matrix–reinforcement phase. The incorporation of plant fibers (e.g., agricultural waste) as a reinforcement in various composites leads toward sustainable development in terms of reducing environmental pollution, conserving natural resources, and improving the economy. These agricultural wastes are otherwise burned, contributing significantly to environmental pollution. Unlike steel/synthetic fibers [47,53,54,55], plant fibers—e.g., hemp, jute, bamboo, kenaf, etc. [38,56,57]—have multiple benefits, such as; low costs and abundant availability. Alyousef et al. [58] reported the same for sheep wool fiber when used as dispersed reinforcement in a cementitious concrete composite to enhance the mechanical properties of said concrete composite. The incorporation of low-density (1.2–1.6 g/cm^3^) plant fibers produces lighter composites compared to synthetic fibers. Accordingly, the demand for composites reinforced with plant fibers (e.g., palm, kenaf, sugarcane, jute, hemp, sisal, coir, banana, etc.) is increasing in the construction industry. However, the durability of plant fibers is still questionable, due to their organic and biodegradable nature, thus restricting their applications as structural/critical materials. The durability of plant fibers and the fiber–matrix interaction are typically optimized by applying chemical treatments to plant fibers.

Hence, it can be said that the potential incorporation of abundantly and locally available plant fibers/agricultural byproducts in different matrices, as reviewed in the present study, can play a significant role in the development of green and sustainable structures. However, as revealed from the literature, the applicability of plant-fiber-reinforced composites is still questionable for structural materials, due to their lesser density affecting their mechanical properties, and their organic/biodegradable nature affecting the long-term durability of the composites. Several studies have been conducted on multiple treatment methods for improving the mechanical properties and the long-term durability of plant fibers and plant-fiber-reinforced composites for use in civil engineering structural applications. Therefore, to summarize the available literature on plant fibers and their composites for construction and building materials covering all of the abovementioned aspects, this review on plant fibers is essential for the development of eco-friendly composites.

## 2. Scientometric Analysis

### 2.1. Methodology

In this study, a scientometric analysis of the literature on plant fibers is carried out to show the error proximity of multiple studies [43,59,60,61]. Scientometrics, if used alone, produces less skewed and more rational results [62,63]. Research over the past two decades is summarized in the present study. Quantitative assessment of research progress is made by using connections and maps among bibliometric data. A compilation of bibliometric data for the analysis of plant fibers was carried out using Scopus. Data refinement techniques were also used. In the specific drop-down menu—i.e., document type—the “review”, “article”, “dissertations”, and “books” options were selected. In addition, for “language”, “English” was chosen. Scientific visualization is used in scientometric reviews, which is a method formulated by researchers for analyzing literature reviews for different purposes [64]. This explains the issues faced by researchers in performing literature reviews manually, and also develops links between countries, authors, sources, articles, and keywords in a specific study area [65]. VOSviewer (version: 1.6.16) was used to create the scientific visualization and mapping. This is an open-source, free visualization tool that is highly recommended in the literature for application in various fields [66,67]. The analysis was performed by utilizing VOSviewer, with “create a map from bibliographic data” as a parameter of “type of data” and “read data from bibliographic database files” as a parameter of “data source”. VOSviewer was applied for importing the CSV files from Scopus. The analysis of all of the frequently appearing keywords, articles, sources, most referenced articles/authors, and regional participation was performed as a step for mapping the review of the science. Maps were utilized to depict different factors, their co-occurrence, and relationships between them, whereas tables were used to summarize the numerical figures.

### 2.2. Scientific Mapping Results and Discussions

#### 2.2.1. Annual Publication Trends

**Figure 4:**
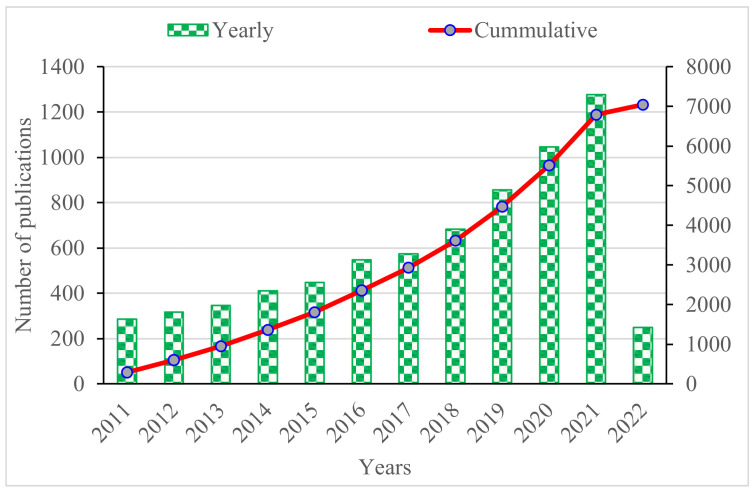
Annually published articles.

The Scopus analyzer was used to analyze the data gathered from its database to assess the research areas with the closest relevancy. It is noteworthy that bibliometric data, starting from 2011, were retrieved by using the time duration limit. Figure 4 shows the annual trends of the publications in the explored research area, from 2011 to 2022 (March). The keywords/terms that we searched in Scopus were “plant fibers”, “applications of plant fibers”, “plant fiber reinforced composites”, “plant fibers building and construction material” and “plant fiber structural and non-structural applications”. It was observed that there was a gradual rise in the number of publications regarding the utilization of plant fibers for different composites. In the same manner, the cumulative publications were also increased. It is an interesting fact that researchers are exploring the potential utilization of plant fibers from various sources for their incorporation in different composites for structural and non-structural applications.

#### 2.2.2. Scientific Mapping of Keyword Co-Occurrence

**Figure 5:**
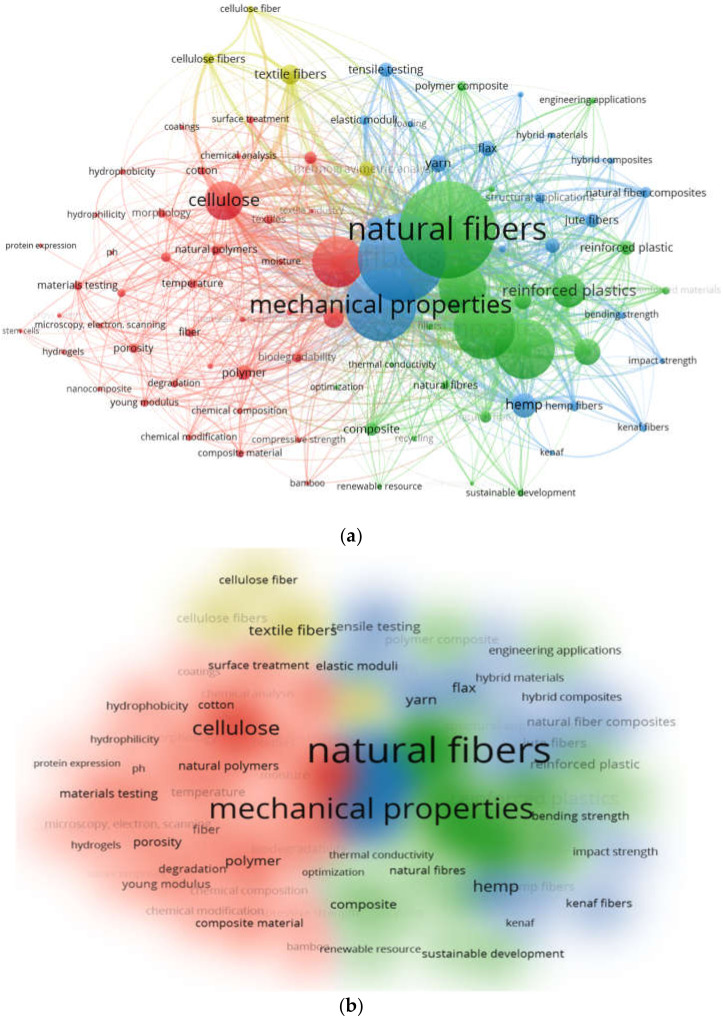
Network based on all keywords: (**a**) occurrence during the last 5 years; (**b**) density showing the recent focus on sustainable development.

**Figure 6:**
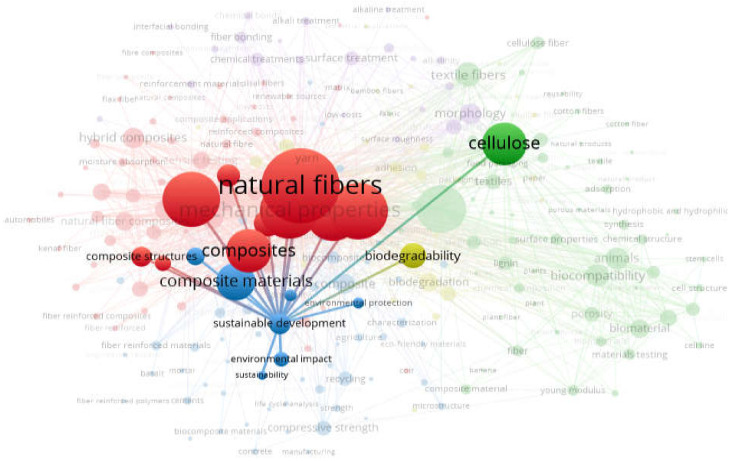
Sustainable development linkage with all factors.

The vital areas of a specific research domain are mainly depicted and indicated by keywords. The most frequently occurring keywords that were used in the present review are discussed in Section 2.1. The co-occurrence network of keywords, along with their visualization, link strength density, and interconnectivity, is shown in Figure 5. The keyword node size depicts a particular keyword’s frequency, whereas co-occurrence of respective publications represents the keyword’s position. The color visualization of the last five years was carried out, such that bigger nodes show a greater number of studies on natural/plant fibers from various sources. Different keywords were bifurcated using distinct/different colors, representing the co-occurrence of keywords in multiple publications. Figure 5a depicts the defined keyword clusters in the following colors: green, blue, red, and yellow. The yellowish tint in the visualization depicts the emerging trend of plant fiber applications in the recent past. The most frequently co-occurring keywords are represented by green nodes, i.e., the composites that are reinforced with plant fibers for various applications. It can be said that all of the abovementioned keywords have repeatedly been utilized in publications regarding different sources of plant fibers. This trend significantly supports the concept of sustainable development, as can also be seen in the density visualization (Figure 5b). The lower- and higher-density keywords are presented with unique colors. The color order is red, depicting the highest density; yellow, moving from the higher side towards the lower one; followed by green and ultimately, blue, depicting the lowest density. This observation could assist in the selection of keywords to conveniently and effectively retrieve the published data on the required domain in future studies. The linkage of all the plant fiber aspects/factors with keywords related to sustainable development is shown in Figure 6. It can be concluded that sustainable development in terms of plant fibers’ applicability is significantly linked with those plant fibers’ properties, chemical composition, bio-degradable nature, related composites, etc.

## 3. Plant Fiber Types and Annual Production

**Figure 7:**
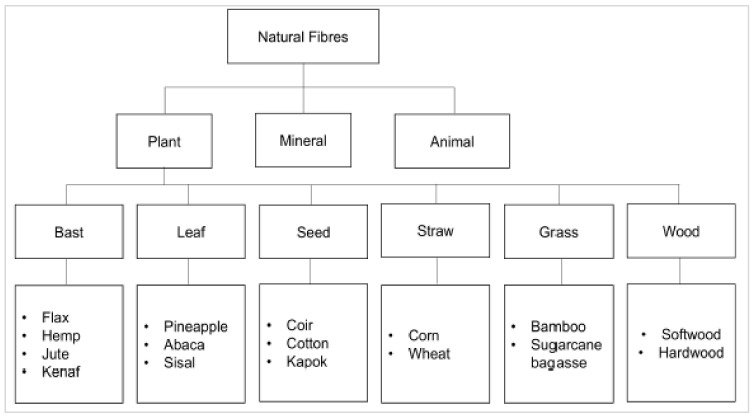
Source-based classification of natural fibers [74].

“Fiber” is a hair-like continuous filament material extracted from an elongated piece, just like a thread, whereas the “fibers” refers to a group of fibers coiled into rope [2,3]. However, they may also be applied in considerably short lengths, or may be ground into powder for application as a filler. Natural fibers are non-manmade and non-synthetic fibers that are extracted from animals or plants. Natural fibers are gaining much attention from researchers for incorporation as dispersed reinforcement in different composites, because of their considerable and comparable mechanical properties, readily abundant availability, flexible handling, and sustainability [68,69,70,71,72,73]. The source-based classification of natural fibers is shown in Figure 7. Among all of these natural fibers, plant fibers are highest in market demand, as they are the most widely used in multiple applications.

Ramamoorthy et al. [75] classified plant fibers into six categories: straw, seed, bast, wood, grass, and leaf fibers. Bast fibers comprise tube-like cell walls, and are extracted from the outermost layers of various plant stems. Multiple industries—particularly the construction industry—incorporate the application of this type of fiber. Furthermore, the need for bast-fiber-reinforced polymer composites is increasing day by day because of their significant properties, such as economy, reliability, non-toxicity, lighter weight, and structural soundness. The extraction from non-fibrous material scrap produces a hard fiber known as leaf fiber. Leaf fiber is coarser and stiffer compared to bast fiber, resulting in its comparatively lesser market demand [76]. The fibers obtained from various plant seeds are named seed fibers. Coconut husk, kapok, and cotton are among the major seed fibers that are incorporated in hybrid natural-fiber-reinforced composites. Similarly, the incorporation of different straw fibers (e.g., rice, rape, barely, wheat, etc.)—due to the abundant production of these crops in various subtropical regions—in different matrices (e.g., soil, straw boards, bales, earthen bricks, brick blocks, mud mortar, cement–sand mortar, lightweight cement walls, etc.), for a number of structural and non-structural applications, has also been studied by many researchers [77,78,79,80]. Due to their lower water content, the applications of straw fibers also include straw-reinforced polymers. In most cases, straws are considered to have less microbial respiration, along with relatively more stability [81]. Therefore, the composites reinforced with straw fibers are excellent. Wheat straw is the product of the wheat crop, and is usually available in surplus to requirements in many countries. Hence, due to its cheap availability and easy access, the use of wheat straw in civil engineering applications is effective [43,44,46,68,82,83].

Fibers extracted from lengthened sclerenchyma cells found in various components of the plant are termed grass fibers. Grass fibers are the same derivatives of synthetic fibers for incorporation in composites. This fiber has been studied for reinforcement in polymer matrices in multiple works aiming to obtain partially biodegradable green composites. In woody plants, there is a fibrous biological tissue that is found in the internal parts of the roots, branches, and stems, and is named wood fiber. As per the taxonomy of plants, woody species are classified into two major types: softwood and hardwood. These are essentially biomaterials with an optimal hierarchy that transfers both external and internal forces, depicting its suitability as the structural material for various applications [84]. The annual production of some natural fibers is given in Table 1.

## 4. Properties of Plant Fibers

### 4.1. Chemical Composition

**Figure 8:**
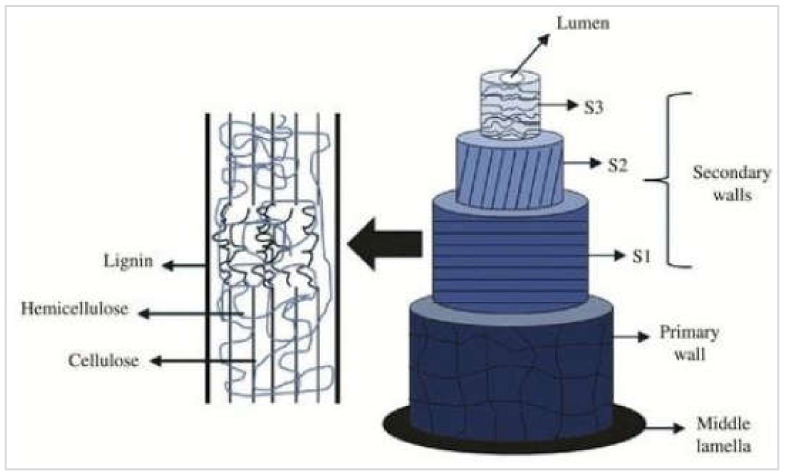
Structure of a plant fiber [120].

**Figure 9:**
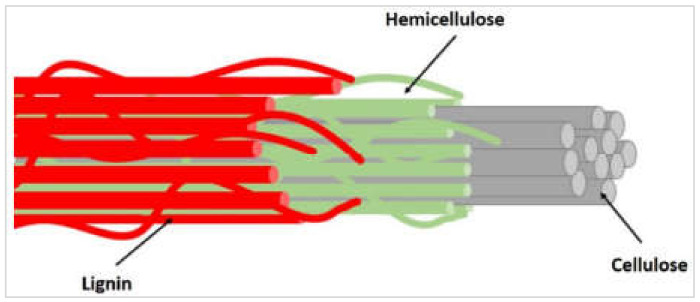
Natural lignocellulosic fiber [74].

Considerable variation in the chemical constituents of plant fibers of diverse types and origins is usually observed [117]. This variation may also be influenced due to growing and harvesting conditions. The lignocellulosic composition of various plant fibers is shown in Figure 8 and listed in Table 1. The lignocellulosic composition of plant biomass mainly comprises lignin ${\left[C_{9}H_{10}O_{3}{\left({\mathrm{OCH}}_{3}\right)}_{0.9-1.7}\right]}_{x}$, cellulose ${\left(C_{6}H_{10}O_{5}\right)}_{n}$, and hemicellulose ${\left(C_{5}H_{8}O_{4}\right)}_{m}$, in the form of strong and complex cellulose–hemicellulose–lignin bonds within a plant [75,118,119]. Figure 9 shows the main structure of a natural lignocellulosic fiber that comprises lignin, hemicellulose, and cellulose, as reported in [74]. Usually, the ranges for lignin, hemicellulose, and cellulose in a conventional lignocellulosic material are 15 to 25%, 30 to 60%, and 20 to 40%, respectively. The mechanical properties of the fibers and their respective matrices are significantly dependent on these lignocellulosic compositions.

**Figure 10:**
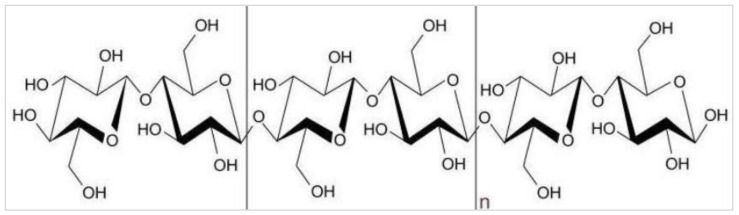
Cellulose structure, i.e., β-D-glucopyranose interlinked with (1–4) glycosidic bonds in a polymer [127].

Cellulose is the main component in biomass, and has several applications in different fields of the industry today [121,122,123,124,125,126]. It is essentially a linear homopolymer with a greater molecular weight that consists of β-D-glucopyranosyl units interlinked with 1–4 glycosidic linkages (Figure 10). It may also be present in anhydroglucose monomer linear chain units, linked with 1-4 linkages, or balanced at the end terminal with reducing and non-reducing sugar units. The cellulose chain characteristics may be assigned to reactive (^−^OH) groups that occupy the C-2, C-3, and C-6 positions. It should be noted here that the ability of such hydroxyl groups to make hydrogen bonds plays an important role in having crystalline packing, and also drives the cellulose’s physical properties. The said interlinked hydrogen bonding of different molecules of cellulose forms microfibers, which interact to make a fiber. Cellulose fibers, due to their biodegradability, low weight, cheap and abundant availability, renewability, and unabrasive nature, as well as their comparable mechanical properties, are being utilized for multiple applications.

Hemicellulose is the second most abundant lignocellulosic constituent, comprising polysaccharide short chains such as galactomannan, xylan, glucomannan, glucuronoxylan, xyloglucan, and arabinoxylan, which are grouped together with β-(1,4) and β-(1,3) glycosidic bonds. The low degree of non-crystallinity and the polymerization nature of hemicellulose cause the disintegration of monosaccharides; therefore, most of its applications are in cosmetics, hydrogels, and drug deliveries. As far as lignin is concerned, it comprises 3D crosslinked polymer with structural units of phenyl propane, and has variations based on the replacement of methoxyl groups with aromatic rings. These are further crosslinked with aryl ether linkages, such as carbon–carbon bonds, β-O-4, and α-O-4, e.g., 5-5, β-β. Guaiacyl (G), p-hydroxyphenyl (H), and syringyl (S) are the three basic constituents of the polymer lignin. The function of lignin is to provide a safeguard by linking covalently with hemicellulose and cellulose, increasing the lignocellulosic biomass recalcitrance.

### 4.2. Physical and Mechanical Properties

**Figure 11:**
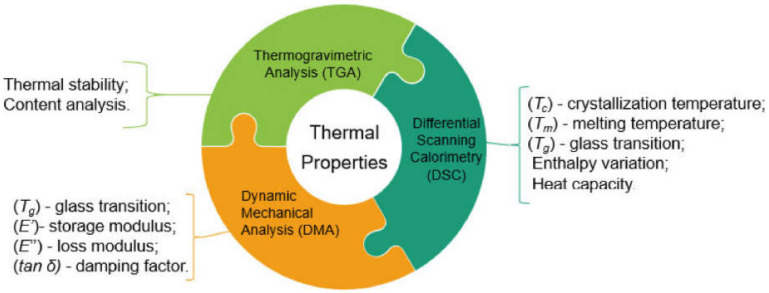
Summary of plant fibers’ thermal evaluation methods [139].

**Figure 12:**
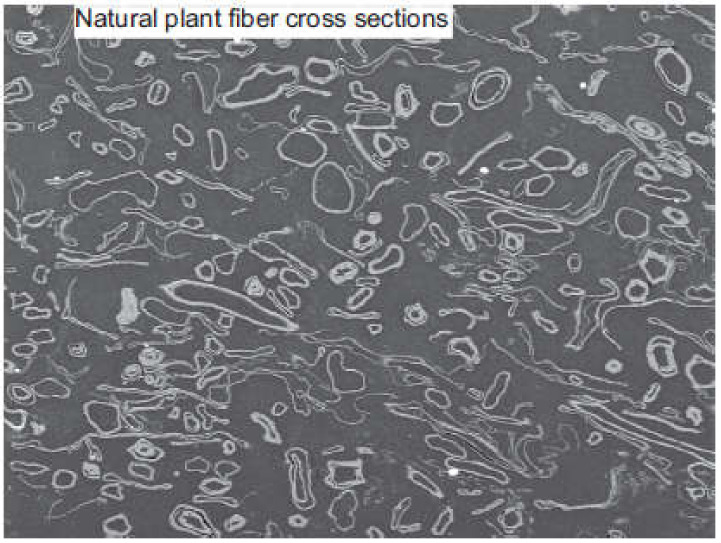
A natural plant fiber cross-section [14].

Some of the physical and mechanical properties of different plant fibers are given in Table 2. The mechanical characteristics of plant fibers are comparatively worse than those of the artificial and synthetic fibers, such as glass fiber, etc. [128]. However, due to the lower density of plant fibers, the physical properties—e.g., strength, property-to-density ratio, and stiffness—of plant fibers are comparable with those of artificial fibers [129,130]. The common geometric properties of plant fibers include their length, diameter/width, and corresponding aspect ratio (Table 2). The fibers’ properties are dimension-dependent. Aspect ratio is one of the governing factors behind their mechanical properties, and can be extracted from geometric classification. A lesser aspect ratio restricts the fibers’ reinforcement capability. The aspect ratio of the fiber in any matrix must be higher than the critical value for having maximum stress transfer to the fiber prior to matrix failure in order to achieve maximum reinforcement. Meanwhile, a lower aspect ratio of the fiber with respect to the critical value leads to inadequate stress transfer, ultimately resulting in poor reinforcement. Doan [131] reported that the fiber length plays an important role in improving fiber-reinforced composites’ mechanical performance. Generally, an increasing trend in the mechanical properties of composites is observed with the increase in the length of the fibers [132]. Baiardo et al. [133] found that the mechanical characteristics of short-fiber-reinforced composites are mainly dependent on (1) fiber aspect ratio, lengthwise distribution, volume, and the fibers’ orientation in the composite; (2) the natural properties of the fibers and matrix, and (3) effective adhesion between the matrix and fibers, which provides for load transfer in the composite. However, a decline in tensile strength was reported upon reducing the fiber length from 9 to 3 mm, due to two major reasons: the existence of gaps, and the weaker bonding between the fiber and the matrix [134]. As far as the effect of the fiber cross-section on the mechanical properties of fiber-reinforced composites is concerned, [135] reported its significant effect on bonding between cementitious matrix and fibers, as well as the flexural toughness of fiber-reinforced composites. Furthermore, in some scenarios, fiber is simply incorporated as a filler [136,137]. Plant-based natural fibers do not cause much damage, as they are nonabrasive due to their biodegradable nature. However, higher water absorption is one of the major hindrances of plant fibers for various applications. This is a basic characteristic of plant fibers because of free hydroxyl along with existing polar groups. It tends to lead to reduced dimensional stability and mechanical characteristics, but it may also act positively towards biocomposites’ biodegradability [138]. In the process of water absorption, the plant fiber cell walls are saturated with water. Furthermore, the void spaces are occupied by water. The water absorption is dependent on various parameters, such as fiber permeability, fiber loading, temperature, surface protection, fiber orientation, void content, exposed surface area, diffusivity, and hydrophilicity [14]. Plant fibers are good thermal and acoustic insulators due to their cellular and hollow nature. Due to this hollow structure, the bulk density of plant fibers is reduced, resulting in their lighter weight. The 40–50% lesser density of plant fibers compared to synthetic fibers is a bonus. The densities of plant fibers vary from type to type. Accordingly, the densities of some plant fibers, along with their respective mechanical properties, are given in Table 1. However, the thermal stability of plant fibers is a matter of concern. Accordingly, limited thermal stability is therefore another challenge for using plant fibers in different composites, as 200 °C is the temperature limit for the processing of plant fibers [105]. Different surface treatments are used to enhance the thermal stability of plant fibers. Various techniques are applied to evaluate the thermal characteristics of plant fiber composites, and to recognize and assess the applicability of various plant fibers for specific applications [5]. The approaches applied in the literature to analyze the thermal stability of plant fibers include differential scanning calorimetry (DSC), thermogravimetric analysis (TGA), and dynamic mechanical analysis (DMA). The main such techniques are summarized in Figure 11, as reported in [139]. In parallel, plant fibers have high stiffness and strength (Table 1). It may be noted that all plant fibers have cellulose fibrils of 10–30 nm in diameter, and consist of up to 30–100 cellulose molecules in a chain conformation that improve the mechanical strength of the fiber. The Young’s modulus and tensile strength of plant fibers are in direct proportion to their cellulose content [14]. As already mentioned, the chemical composition of plant fibers consists of lignin, cellulose, hemicelluloses, waxes, and pectin. Figure 12 shows the scanning electron microscope (SEM) image of a cross-section of plant fiber. In plant fibers, the reinforcing components are cellulose microfibrils, and these microfibrils are surrounded by lignin and hemicelluloses. Upon application of loading, these microfibrils are in line with the axis of the fiber. The fiber failure occurs because of breakage in hydrogen bonds due to the loss of bonding between matrix elements and reinforcing fibrils. Accordingly, the lower the cellulose content of a plant fiber, the lower its tensile strength [140]. The plant fiber stiffness is determined by its cellulose fibrils’ orientation with respect to the fiber axis. The spiral orientation of fibrils with respect to the fiber axis results in the ductility of the plant fibers. Meanwhile, the higher tensile strength and rigidity of plant fibers are due to the parallel orientation of fibrils with respect to the fiber axis [141,142]. It should be noted here that the physical/mechanical properties of plant fibers are origin- and climate-dependent. However, plant fibers with poor mechanical properties may also be utilized in non-structural applications.

The fracture strain, also known as elongation at break, is the ratio of change in length to the original length after the specimen is broken. It shows the plant fiber’s ability to resist the change in shape to avoid crack formation by providing a bridging mechanism. EN ISO 527 is the test standard for the determination of elongation at break. Usually, the physical and mechanical properties of synthetic fibers are better than those of plant fibers. However, in the case of elongation at break and specific modulus, plant fibers are better. Fibers from leaf and bast possess low elongation at break compared to stalk or seed fibers. Elongation at break values for various plant fibers are summarized from the literature in Table 1. The capability of plant fibers to bear the bending load that is applied perpendicular to their longitudinal axis is called flexural strength. In this scenario, the plant-fiber-reinforced beam composites are more appropriate. In this way, the higher fiber content means greater modulus and flexural strength. Long plant fibers—such as bast and leaf fibers—have the highest efficiency among the lignocellulosic reinforcements. The fiber length is a key factor to improve the fracture toughness and flexural strength of composites incorporating plant fibers.

## 5. Treatment Methods of Plant Fibers

**Figure 13:**
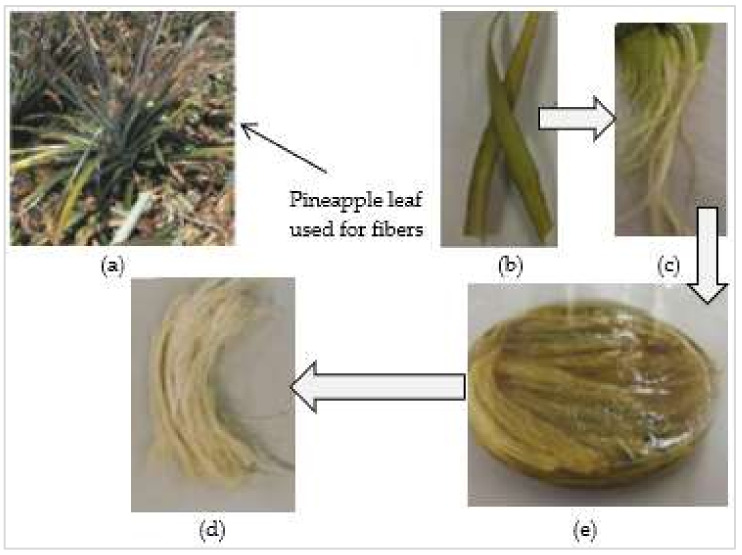
Pineapple fiber: (**a**) plant, (**b**) leaves, (**c**) raw fiber, (**d**) 1% NaOH treatment solution, and (**e**) treated fibers [152].

In addition to enhancing the mechanical properties of composites by incorporating plant fibers, their durability should also be given proper consideration due to the biodegradable nature of these fibers. Deficiencies/degradation of plant-fiber-reinforced composites, in terms of durability, are usually observed due to their organic nature. This might be due to the mineralization of fibers and alkaline attacks under exposure to climatic conditions [148,149]. The complex microstructural heterogeneity and high water absorption of plant fibers also affect the properties of their composites. Furthermore, the pectin and waxes present in the cell walls of plant fibers prevent the interlocking within a matrix. Accordingly, this leads to poor adhesion of the fiber with the matrix, poor strength properties, and weak dispersion of force. The structural composition of plant fibers—i.e., lignin, cellulose, hemicellulose, wax, and pectin—shows an unsteady effect due to moisture and weak adhesion with the surrounding matrix [150,151]. Hence, there is a need to modify/improve the plant fibers’ properties to overcome the deficiencies associated with them. The extraction of one plant fiber (i.e., pineapple) from the respective plant in a raw and chemically treated form is shown in Figure 13, as presented by Putra et al. [152]. These alterations in plant fibers are intended to modify the chemical, physical, or morphological properties of the fibers, or to safeguard the natural hydrophilic fibers against proper bonding with the surrounding matrix. The main purpose of chemical treatments for plant fibers is essentially to improve the fibers’ properties by modifying their microstructure in parallel with enhancement of their surface morphology, chemical groups, tensile strength, and wettability [153,154]. Multiple techniques—i.e., chemical, biological, and physical—have been proposed. Among these, some treatment techniques for different plant fibers are summarized from the literature in Table 3. Multiple treatments—including benzoyl chloride, alkalis, acetic anhydride, potassium permanganate, acetic acid, silane, and peroxides—are used to treat plant fibers. As reported in the literature [155], these treatment techniques are intended for improving the plant fibers’ mechanical properties by modifying their crystallinity and eliminating the weaker constituents—i.e., fats, lignin, and pectin—from the surfaces of the fibers. Furthermore, as a result of chemical treatment, the structural components with partial cementing are split and removed, providing a rough and clean fiber structure. This rough surface of plant fibers improves the bonding mechanism of the fiber with the surrounding matrix, thus enhancing the mechanical properties of the composite [155].

**Figure 14:**
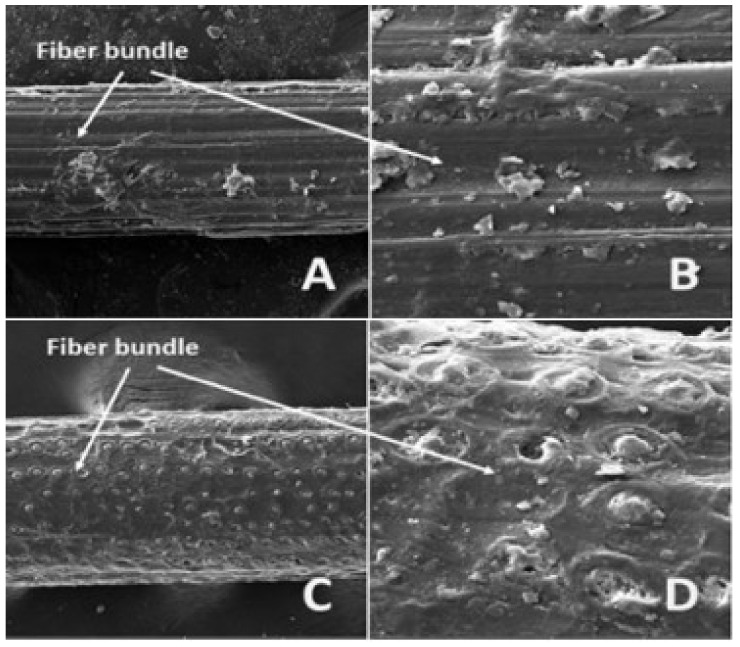
SEM images of untreated abaca and coir fibers: (**A**) abaca fiber bundle (@ mag 750 x), (**B**) abaca fiber detailed surface view (@ mag 3.5 kx), (**C**) coir fiber bundle (@ mag 750 x), and (**D**) coir fiber detailed surface view (@ mag 3.5 kx) [193].

**Figure 15:**
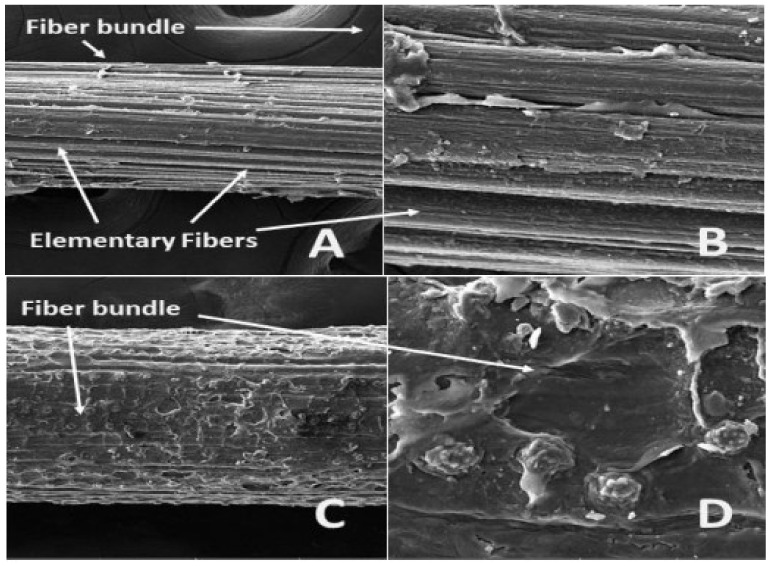
SEM images of 5%-NaOH-treated abaca and coir fibers: (**A**) abaca fiber bundle (@ mag 750 x), (**B**) abaca fiber detailed surface view (@ mag 3.5 kx), (**C**) coir fiber bundle (@ mag 750 x), and (**D**) coir fiber detailed surface view (@ mag 3.5 kx) [193].

**Figure 16:**
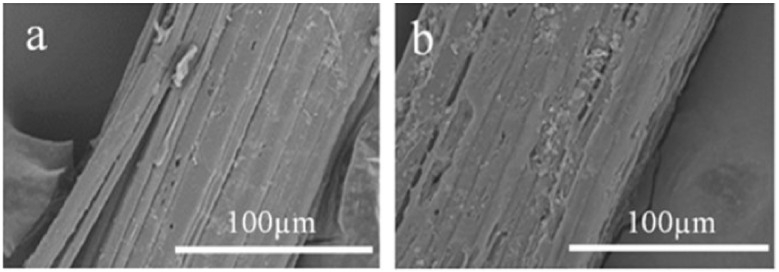
SEM images of alkali-treated jute fiber: (**a**) raw, (**b**) treated [194].

Tserki et al. [190] explored the impact of acetylation on wood, hemp, and flax fibers. The removal of non-crystalline fractions from fibers was observed after this treatment. These altered surface properties led to improvements in interface stress transfer characteristics. Hossain et al. [191] also observed similar behavior upon application of alkaline treatment of ladyfinger fiber. Latiff [192] also reported a 47.5% improvement in the tensile strength of fibers upon soaking in benzoylation treatment for 30 min. The SEM images of two alkaline-treated and -untreated plant fibers (i.e., abaca and coir) are shown in Figure 14 and Figure 15, respectively. Similarly, the SEM images of untreated and treated jute fiber are shown in Figure 16. Hence, it can be concluded that a considerable enhancement in the mechanical properties of plant fibers and their respective matrices can be attained by applying chemical treatments.

## 6. Application of Plant Fibers as Construction and Building Materials

**Figure 17:**
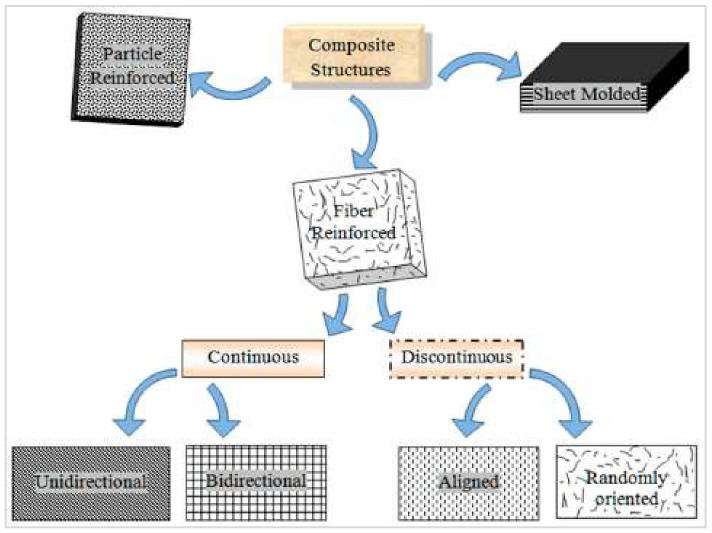
Classification of plant-fiber-reinforced composites [52].

The structural and non-structural applications of plant fibers are increasing expeditiously in multiple fields of engineering. Plant fibers have been incorporated as reinforcement in various composites, including sugarcane [195,196], hemp [196], corn [197], kenaf [198,199,200,201,202], ramie [94], water hyacinth [203], flax [204], ginger [205,206], coir [207], cotton [208,209], sisal [210], banana [211], oil palm [212,213], sugar palm [214,215], and wood [108]. Figure 17 shows a broader classification of plant-fiber-reinforced composites.

In addition to biodegradability, there are other several benefits of plant fibers, including easy availability, the substitution of timber plastic composites, low cost, and reduced deforestation [216]. Plant fibers can potentially be utilized in multiple composites [217]. Ilyas et al. [218] reported plant fibers as an alternative material to carbon and glass fibers. Plant fibers—such as hemp, oil palm, jute, curauá, bamboo, and kenaf—when incorporated in different composites, have multiple applications in the construction industry [43,60,68,73,153,219,220,221]. In addition, the plant fibers can also be used as potential materials for insulation, acoustic, and architectural applications, i.e., subtypes of construction and building materials [18,19,20,21,22,23,24,25]. Plant fibers are considered to be more appropriate materials to meet the needs of the modern era, such as three-dimensional flexibility and forest management, while achieving a building that is both functional and aesthetically pleasing. It should also be mentioned here that, due to the availability of plant fibers in a wider variety range, their applications may also lead towards novel creative methods of enhancing the interaction of people with surrounding spaces.

The properties of plant-fiber-reinforced cementitious composites are comparable to those of steel and artificial-fiber-reinforced composites for applications in civil engineering [130,168,222]. As already mentioned, significant interest has been established in the past few years towards incorporating plant fibers in cementitious composites to obtain alternative economical, eco-friendly, and sustainable construction and building materials. Plant fibers can potentially be incorporated as dispersed reinforcement in concrete to overcome concrete’s traditional deficiencies. The enhancement in the energy-absorbing capacity of brittle concrete can be achieved by using plant fibers in it [168,223,224]. Researchers have used plant fibers—such as banana, vakka, wheat straw, ramie bast, pineapple leaf, jute, abaca leaf, kenaf bast, flax, coir, palm, hibiscus cannabinus, elephant grass, bamboo, malva, sisal, guaruman, sansevieria leaf, piassava, hemp, sugarcane, and date—as dispersed reinforcements in cementitious composites for different civil engineering applications, as an alternative replacement for artificial/steel fibers [43,183,225,226,227,228,229,230,231,232,233,234,235,236,237]. Ali et al. [66] evaluated the dynamic and mechanical properties of coconut-fiber-reinforced concrete for possible application in earthquake-resistant housing. Terai and Minami [230] experimentally determined the shear and flexural properties of bamboo-reinforced cementitious composites. The fracture energy of elephant-grass-, hemp-, and wheat-straw-reinforced concrete was evaluated by Merta and Tschegg [238]. Wheat-straw-reinforced mortar and concrete were explored by Albahttiti et al. [239] and Farooqi and Ali [43], respectively. Hence, it can be said that plant fibers have significant potential to be used as an alternative construction and building material.

### 6.1. Mechanical Properties of Plant-Fiber-Reinforced Cementitious Composites

#### 6.1.1. Compressive Strength

**Figure 18:**
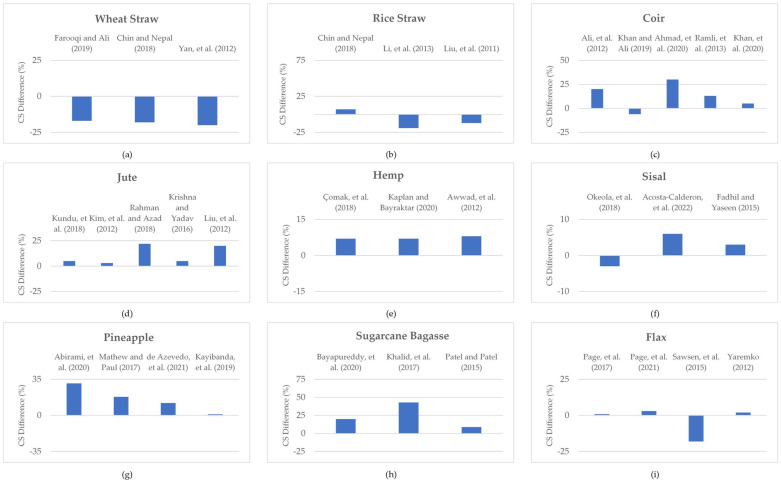
Percentage difference in compressive strengths of (**a**) Wheat straw, (**b**) Rice straw, (**c**) Coir, (**d**) Jute, (**e**) Hemp, (**f**) Sisal, (**g**) Pineapple, (**h**) Sugarcane Bagasse, and (**i**) Flax fiber reinforced cementitious composites with respective reference composites.

The aim of using plant fibers as alternative and sustainable construction and building materials leads towards their application as dispersed fibers in cementitious composites. Remarkable studies have been conducted to experimentally determine the mechanical properties of various plant-fiber-reinforced cementitious composites. Accordingly, the compressive strengths of different plant fibers have been reported in the literature. The percentage differences in the compressive strength of some plant-fiber-reinforced cementitious composites with respect to reference/control composites are shown in Figure 18. The percentage difference in the compressive strength of wheat-straw-reinforced concrete compared to that of plain concrete is shown in Figure 18a, as reported in the literature [43,79,240]. A decrease in compressive strength is observed upon the incorporation of wheat straw in concrete. Similarly, the percentage difference in the compressive strength of rice-straw-reinforced concrete is presented in Figure 18b. Although Chin and Nepal [79] reported an enhancement of up to 7% in the compressive strength of rice-straw-reinforced cementitious composites, at the same time, decreases in the compressive strength of said composites were also reported by Li et al. [241] and Liu et al. [242]. As far as the incorporation of coconut fibers in cementitious composites is concerned (Figure 18c), most studies have reported enhanced compressive strength [60,243,244,245], whereas Khan and Ali [246] reported a slightly reduced compressive strength of coconut-fiber-reinforced concrete. Usually, enhanced compressive strength is reported upon the addition of jute fiber to cementitious composites (Figure 18d) [247,248,249,250,251]. In the same manner, as shown in Figure 18e, the addition of hemp fibers to cementitious composites also usually results in an increase in compressive strength [252,253,254]. Figure 18f shows that the compressive strength of sisal-fiber-reinforced concrete is more or less same (i.e., from 97% to 106%) as that of control specimens [255,256,257]. The percentage differences in the compressive strength of pineapple, sugarcane bagasse, and flax fibers are shown in Figure 18g–i, respectively. In most of the reported studies, enhancement in the compressive strength was observed with respect to reference composites [83,258,259,260,261,262,263,264,265,266]. However, Sawsen et al. [267] reported reduced compressive strength for flax-fiber-reinforced concrete. The addition of plant fibers is primarily considered for increasing compressive toughness instead of compressive strength. The performance of a structure cannot be gauged by its compressive strength only, as its toughness parameter also contributes to its overall performance [268,269]. Hence, the dispersed plant fibers are usually incorporated in brittle cementitious composites to increase their toughness with minimal or no loss of compressive strength [270]. The enhanced compressive strength in the case of some plant-fiber-reinforced cementitious composites is an added bonus.

#### 6.1.2. Flexural Strength

**Figure 19:**
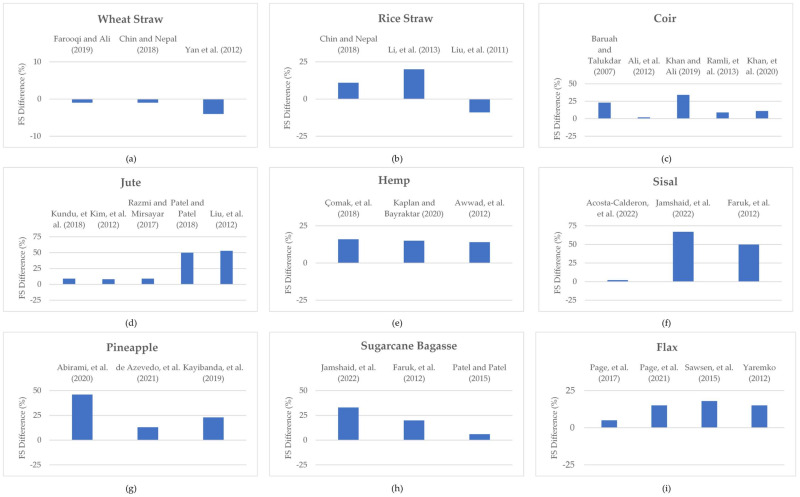
Percentage difference in flexural strengths of (**a**) Wheat straw, (**b**) Rice straw, (**c**) Coir, (**d**) Jute, (**e**) Hemp, (**f**) Sisal, (**g**) Pineapple, (**h**) Sugarcane Bagasse, and (**i**) Flax fiber reinforced cementitious composites with respective reference composites.

The mechanical properties of plant-fiber-reinforced cementitious composites in terms of flexural strength have also been investigated for a variety of applications in construction and building materials. Accordingly, the percentage differences in the flexural strengths of various plant-fiber-reinforced cementitious composites, as extracted from the literature, are shown in Figure 19a–i. It may be observed from the reported studies that wheat-straw-reinforced cementitious composites have more or less similar flexural strength to that of control composites [43,79,240]. However, enhanced flexural strength has been reported in literature for rice-straw-reinforced cementitious composites [79,242,271]. In contrast, Li et al. [241] reported reduced flexural strength of rice-straw-reinforced concrete compared to that of plain concrete. In the case of the flexural strength of coconut-fiber-reinforced cementitious composites, most studies have reported increased flexural strength [243,244,245,246,272]. In a similar way, the majority of studies have reported enhancements in the flexural strength of jute- [247,248,251,273,274] (Figure 18d), hemp- [252,253,254] (Figure 18e), sisal- [4,256,275] (Figure 18f), pineapple- [83,258,260] (Figure 18g), sugarcane-bagasse- [4,263,275] (Figure 18h), and flax- [264,265,266,267] (Figure 18i) fiber-reinforced cementitious composites, compared to control specimens/composites. Overall, it may be concluded that, in comparison to compressive strength, the effect of fiber is more significant with respect to enhancements in the flexural strength of cementitious composites. In the case of airport and road pavement applications, the flexural strength is a governing parameter [276]. Hence, the reported enhancements in the flexural strength of most of the plant-fiber-reinforced cementitious composites may significantly contribute to the structural performance of pavements [277]. However, the hybrid effect of plant fibers with natural mineral fibers will be an interesting area to explore for civil engineering applications, because previous studies [278,279,280,281,282,283,284,285,286] have reported the enhanced mechanical properties of concrete with the use of natural mineral fibers.

## 7. Conclusions

The present study was intended to perform a scientometric analysis and a comprehensive review of the types, properties, treatment methods, and applications of plant fibers as a step in pursuit of sustainable development. A scientometric analysis was performed on the bibliometric data of the last decade (2011–2022) extracted from the Scopus database, and was analyzed by VOSviewer to evaluate the co-occurrence of keywords in the field of natural fibers. Furthermore, the aspects of sustainability in terms of using plant fibers in construction and building materials, along with moving a step towards reducing landfill pollution, were also discussed. The conclusions of the conducted study are as follows:

- Scientometric analysis revealed an emerging trend of plant fibers for cementitious composites, with a considerable rise in the last five years. Furthermore, it was found from the analysis that there is a strong linkage of plant fiber keywords with sustainability, sustainable development, and environmental impact. Hence, it can be said that multiple techniques to reduce environmental degradation by using plant fibers are under consideration today. In this scenario, the interest in the usage of ecologically and environmentally friendly plant fibers and composites has been steadily increasing over the last decade. Their excellent specific properties, environmental advantages, multiscale structure applications, abundant availability, low cost, and technical feasibility are among the reasons behind the popularity that they have gained.
- The plant fibers that are most commonly incorporated in various composites are coir, flax, jute, hemp, and wheat straw, while sugar palm, roselle, and kenaf are emerging fibers due to their high stiffness and mechanical strength, which make them appropriate for multiple applications in the civil engineering field. Generally, the composition of plant fibers is lignin, cellulose, hemicellulose, and pectin. As reported by several researchers, cellulose is the key factor behind the appreciable mechanical properties of plant fibers, as cellulose provides good structural integrity and shape to the fibers. The facial interaction of plant fibers with the surrounding matrix, due to their smaller particle size, enhances the reinforcement effectiveness to a greater extent. However, the structural applications of plant fibers are still quite limited due to the poor fiber–matrix adhesion and low moisture resistance. These limitations of plant fibers can be eliminated by chemical treatments such as alkalization, benzoylation, silane, and acetylation treatment. Among these, alkali treatment has emerged as an effective and economical method.
- Plant-fiber-reinforced composites have several major applications as construction and building materials, including earthquake-resistant housing, bridge piers, canal linings, soil reinforcement, pavements, etc. The mechanical properties of various plant-fiber-reinforced cementitious composites in terms of compressive and flexural strength have been reported in several studies as being improved by up to 43% and 67%, respectively, with respect to reference composites.
- Processing of natural resources by consuming fossil fuels to meet the construction industry’s needs leads to environmental degradation. Furthermore, agricultural/plant waste burning is also a major contributor to air/environmental pollution. Heading towards sustainable development, the incorporation of plant fibers—e.g., agricultural waste/byproducts—as an alternative to synthetic fibers for reinforcement in different composites can play a significant role in sustainable development by reducing landfill pollution. However, the long-term performance of plant fibers (e.g., agricultural waste) and their reinforced composites is questionable, due to their organic nature.

## 8. Future Recommendations

After conducting a detailed scientometric review of the utilization of plant fibers for sustainable development, we noted that the available research is not sufficient to enable the practical implementation of plant fibers for structural applications in the construction industry. Depending on the advantages of plant fiber applications, it is recommended to conduct a detailed investigation to explore their potential for civil engineering structural applications.

Furthermore, it should be noted here that the durability of plant fibers is a matter of concern. The durability of plant fibers, along with the alkaline nature of cementitious composites, does not provide much hindrance to their use in structural applications. Therefore, the durability of plant fibers must also be given proper consideration, due to their organic nature. Accordingly, more effective pretreatment techniques with a lower environmental impact need to be explored for enhancing the application of plant fibers in construction (structural members).

In general, short discrete fibers, regardless of type and/or source, can be added to concrete to enhance the tensile strength and ductility performance of concrete composites. Therefore, further research should also be carried out regarding the development of optimized design methods that enhance the plant-fiber-reinforced composites’ ductility and strength (e.g., compressive, splitting–tensile, flexural, and shear strengths), manufacturing techniques, and applicability for the construction industry. Perhaps future research focusing on the development of computational techniques should also be carried out to cover the research gap caused by the progressing growth in computational solutions to address complex problems. The development of optimization algorithms for extracting the optimal parameters and design for experimental techniques to conduct variation analysis should also be explored in detail for the reduction in the consumption of time and cost.

Furthermore, future research should also be carried out to explore new composites with combinations of different fibers (i.e., natural and synthetic)—i.e., hybrid fiber-reinforced composites—and the adoption of new methods for the manufacturing of said composites. Last but not least, the life-cycle assessment (LCA) for plant fibers’ development would also benefit the long-term sustainable growth of plant-fiber-reinforced composites’ applications.

## Figures and Tables

**Table 1:** Reviews of the chemical/physical/mechanical properties, chemical treatments, annual production, and approximate cost of plant fibers.

Plant Fiber	Source	Chemical Composition				Physical/Mechanical Properties				Reference	Annual Production		Approx. Cost	
		Lignin	Cellulose	Hemi–Cellulose	Crystallinity	Tensile Strength	Tensile Modulus	Density	Elongation at Break		Production	Reference	Price per Ton	Reference
		Wt.%	Wt.%	Wt.%	Wt.%	MPa	GPa	g/cm^3^	%	–	(×10^3^)	–	USD	–
Bamboo	Stem	10.2	73.8	12.5	40–60	140–230	11–17	1.25	2	[14,74,85,86,87]	10,000	[88,89]	500	[89,90]
Sugarcane Bagasse	Stem	25–32	32–34	19–24	76	290	17	1.5	1–3	[74,86,91,92]	–	–	–	–
Hemp	Stem	3–5	70–92	18–22	50–90	690	70	1.48	1.6–4	[4,14,27,57,74,93,94,95]	215	[88,89]	1550	[89,90]
Kenaf	Stem	15–19	44–87	22	48.2	215	53	1.45	1.6		770		400	
Jute	Stem	5–13	51–84	12–20	50–80	393–773	15.4–26.5	1.3	1.5–2.5		2500		950	
Ramie	Stem	0.6–1	68–76	13–15	55.5	560	24.5	1.5	2.5–3.8		100		–	–
Flax	Stem	2.2–5	60–81	14–20.6	50–90	345–1035	27.6	0.6–1.1	2.7–3.2		810		3150	[89,90,96]
Sisal	Leaf	8–11	67–78	10–14.2	50–70	347–700	9.4–22	1.03–1.5	2–2.5	[74,95,97,98,99,100]	380		650	
Coir	Fruit	40–45	32–43	0.15–0.25	27–33	139	4–6	1.2	30	[74,95,101]	100		–	–
Pineapple	Fruit	4.2	66.2	19.5	35.9	400–627	1.44	0.8–1.6	14.5	[74,102,103]	–	–	455	[89,90]
Cotton	Seed	–	–	–	–	287–587	5–12	1.5–1.6	7–8	[104,105,106,107]	18,500	[88,89,108]	–	–
Wood	Stem	–	–	–	–	–	7–70	0.5–1.4	–	[105,109]	1,750,000		–	–
Wheat Straw	Stem	18.9–25.1	43.1–44.7	32.9–35.3	57.5	21.2–40	4.76–6.58	0.02–0.11	5.4	[110,111,112]	731,460	[113,114,115,116]	60	[116]

**Table 2:** Plant fibers’ properties.

Plant Fibers	Geometric Dimensions			Mechanical Properties	
	Mean Length	Mean Width	Aspect Ratio	Stiffness	Ultimate Stress
	(mm)	(μm)	(-)	(GPa)	(MPa)
Bamboo	2.7	14	193	-	-
Sugarcane Bagasse	0.68–1.7	20–22.8	29.8–85	-	-
Jute	2	20	100	20–55	200–500
Hemp	25	25	1000	30–60	300–800
Coir	0.7	20	35	-	-
Wheat Straw	15	15	100	-	-
Ramie	12–15	20–75	2000–6000	-	-
Cotton	25	20	1250	-	-
Sisal	3	20	150	9–22	100–800
Kenaf	5	21	238	-	-
Flax	33	19	1737	50–70	500–900
References	[14,143,144,145,146]			[14,147]	

**Table 3:** Different proposed treatments for plant fibers.

Plant Fibers	Treatment Techniques	References
Bamboo	Acetylation, potassium permanganate, fiber hybridization	[56,156,157]
Sugarcane Bagasse	acetylation, alkali, stearic acid, fiber hybridization, hydrogen peroxide	[153,158,159,160]
Hemp	Potassium permanganate, nanoparticle grafting	[161,162]
Kenaf	Alkali, nanoparticle grafting, fiber hybridization	[163,164,165,166]
Jute	Alkali, benzoylation, sodium bicarbonate, fiber hybridization, water immersion, nanoparticle grafting	[153,159,166,167,168,169,170]
Ramie	Steam blasting, nanoparticle grafting, silane	[171,172,173]
Flax	Silane, nanoparticle grafting, fiber hybridization	[174,175,176]
Sisal	Alkali, acetylation, nanoparticle grafting, water immersion	[168,177,178,179,180]
Coir	Alkali, permanganate, fiber hybridization, water immersion	[168,181,182,183,184]
Pineapple	Alkali, fiber hybridization	[185,186]
Cotton	Silane, surface fibrillation, nanoparticle grafting	[120,187,188]
Wheat Straw	Alkali, boiling, fiber hybridization, water immersion, nanoparticle grafting	[43,44,46,184,189]

## Data Availability

All data are available in the paper.
